# Lipid-sensor FFAR1 curbs autophagy and mitochondrial respiration in murine retinal angiomatous proliferation

**DOI:** 10.1080/27694127.2023.2177932

**Published:** 2023-02-21

**Authors:** Émilie Heckel, Jean-Sébastien Joyal

**Affiliations:** a Department of Pharmacology and Physiology, Université de Montréal, Montreal, QC, CA, H3T 1J4; b Department of Pharmacology and Therapeutics, McGill University, Montreal, QC, CA, H3G 1Y6; c Department of Pediatrics, CHU Sainte-Justine, Université de Montréal, Montreal, QC, CA, H3T 1C5; d Department of Ophthalmology, Université de Montréal, Montreal, QC, CA, H3T 1J4

Mechanisms driving pathological angiogenesis in neovascular age-related macular degeneration (nvAMD) remain ill-understood. In a murine model of retinal angiomatous proliferation (RAP), a common subtype of nvAMD, we show that excess triglyceride-derived fatty acids are sensed by FFAR1 (free fatty acid receptor 1) in photoreceptors. FFAR1 suppresses TFEB (transcription factor EB), a master regulator of macroautophagy/autophagy and lipid metabolism, contributing to photoreceptors’ energy failure in murine RAP. The metabolic signature observed in murine RAP mimics that of human nvAMD vitreous. Improving autophagy and mitochondrial respiration by deleting *Ffar1* or using autophagy agonists reduces pathological RAP-like lesions. Our findings offer a putative mechanism by which elevated circulating triglyceride-derived fatty acids can dysregulate autophagy and energy homeostasis in photoreceptors and drive pathological angiogenesis.

Neovascular AMD (nvAMD) is the leading cause of blindness in older adults. Retinal angiomatous proliferation (RAP) accounts for 12-15% of nvAMD and is a significant cause of blindness. In RAP, aberrant neovessels originate from the inner retina and invade photoreceptors disrupting vision. We previously showed that nutrient-starved photoreceptors drive pathological angiogenesis in a murine RAP model to reinstate retinal energy homeostasis. Metabolically active cells, such as photoreceptors, rely partly on autophagy to palliate nutrient scarcity. We, therefore, explored the hypothesis that autophagy can help rescue the energy crisis of photoreceptors in a murine RAP model and how autophagy might be dysregulated in that disease.

Conserved across species, autophagy, or the process of ‘self-eating’ by lysosomes, is tasked with clearing defective proteins and organelles and recycling them in part to produce fuel for mitochondria. Ensuring cellular quality control by autophagy is critical in post-mitotic neurons of the retina. Essential functions of the visual system depend on autophagy. In the retinal pigment epithelium (RPE), autophagy recycles visual cycle retinoids from the daily shedding of photoreceptor outer segments to maintain vision. Autophagy also helps prune embryonic vestiges during retinal development. Conditional loss of autophagy in murine photoreceptors disrupts mitochondrial volume and shape, leading to cell demise and vision loss. In developing chick retinas, blocking autophagy reduces ATP levels. Autophagy might therefore contribute to the energy supply of photoreceptors.

Retinal photoreceptors are fuelled by fatty acids (FA) and glucose, which are metabolized to acetyl-CoA to power the Krebs cycle and generate energy. FA β-oxidation (FAO) is an efficient alternative source of energy to glucose in organs with high metabolic rates, such as the heart, muscles, and photoreceptors, tissues that also express VLDLR (very low-density lipoprotein receptor) to facilitate FA uptake. VLDLR anchors chylomicrons and very low-density lipoproteins, locally enabling the cleavage of long-chain FA from triglycerides by LPL (lipoprotein lipase). Indeed, *vldlr* deletion prevents efficient lipid uptake and FAO in the heart and photoreceptors. Functional FAO enzymes are expressed in Müller glia, RPE, and photoreceptors of the lipid-rich retina. Lipid β-oxidation is a critical yet understudied metabolic pathway in the eye, particularly in photoreceptors. *vldlr* knockout (^*-/-*^) mice form RAP-like neovascular lesions invading nutrient-starved photoreceptors from the inner retina, resembling retinal angiomatous proliferation (RAP) in humans. Decreased lipid uptake in *vldlr^−/−^* mice causes an intracellular lipid deficiency and more circulating FA.

Nutrient sensors for glucose and amino acids regulate autophagy. However, the role of lipid sensors in controlling autophagy has been less explored. Fatty acid G-protein coupled receptors are a family of low-sensitivity plasma membrane FA sensors. FFAR1 is highly expressed in photoreceptors and is activated by micromolar concentrations of medium and long-chain FAs, uniquely suited to detect surges of extracellular lipids, such as following a lipid-rich meal, but not basal or starvation FA levels. We recently showed that abundant circulating lipids in the *vldlr^−/−^* RAP model triggers a satiety signal in photoreceptors through FFAR1 that restrains autophagy and oxidative mitochondrial metabolism when nutrient supply exceeds the metabolic needs of photoreceptors [1].

Lipid metabolism and autophagy are transcriptionally co-regulated. TFEB governs lysosomal biogenesis, the expression of crucial FAO enzymes, as well as PPARA/PPARα and PPARGC1A/PGC1α, which are part of its transcriptional network. TFEB is inactive when phosphorylated, preventing its nuclear translocation. In the presence of nutrients, MTORC1 forms a kinase complex that phosphorylates TFEB, inhibiting autophagy. Nutrient scarcity releases TFEB from that complex allowing PPP3/calcineurin, a calcium-dependent phosphatase highly expressed in photoreceptors, to dephosphorylate TFEB, activating its transcriptional program. PPP3/calcineurin regulates TFEB activity downstream and sometimes independently of MTORC1. We recently showed that excess lipids sensed by FFAR1 inhibit PPP3/calcineurin, thereby suppressing TFEB activity and autophagy in photoreceptors. More importantly, *ffar1* deletion in the *vldlr^−/−^* RAP model rescues PPP3/calcineurin and TFEB activity and improves retinal autophagy. Hence, the lipid sensor FFAR1 suppresses autophagy by inhibiting PPP3/calcineurin and TFEB activity^1^

**Figure 1.**
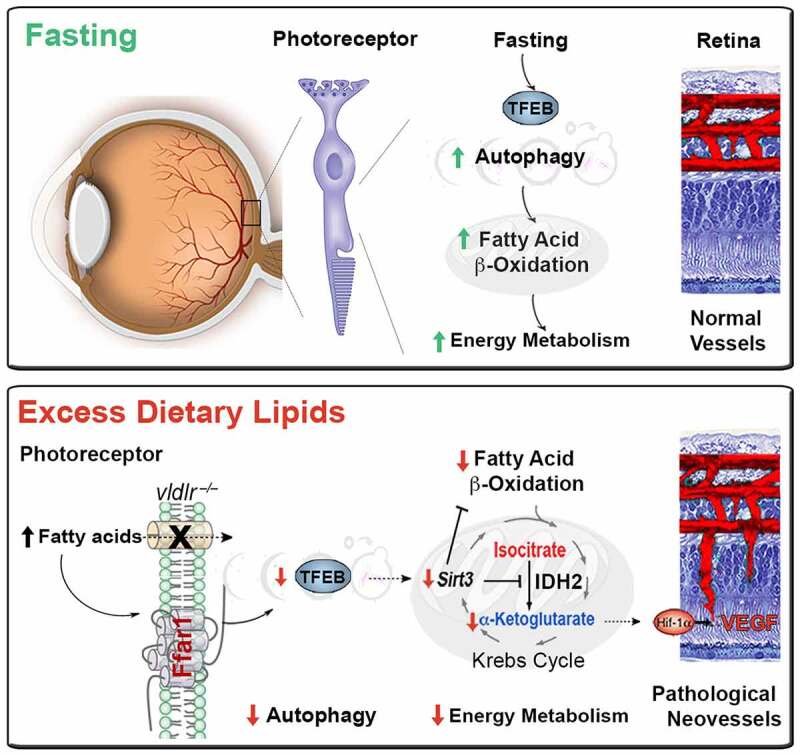
Dysregulated autophagy by triglyceride-derived FAs contributes to the energy failure of photoreceptors and drives neovascular retinal diseases. Fasting activates TFEB and SIRT3, increasing autophagy and mitochondrial efficiency to meet photoreceptors’ energy demands. Conversely, excess lipids in the murine vldlr^−/−^ RAP model suppresses TFEB activity, and SIRT3 expression, contributing to the energy failure of photoreceptors and VEGF secretion driving RAP-like neovascular disease.

TFEB governs lipid metabolism and the expression of PPARGC1A, which plays a central role in cellular energy metabolism. Among many of its functions, PPARGC1A promotes the transcription of SIRT3 (sirtuin 3), an NAD^+^ deacetylase activated by starvation. SIRT3 also promotes mitochondrial FAO that feeds the Krebs cycle. Key enzymes of FAO and the Krebs cycle, such as IDH2 (isocitrate dehydrogenase 2 (NADP+), mitochondrial), are deacetylated by SIRT3. IDH2 catalyzes the conversion of isocitrate to α-ketoglutarate (α-KG). Finally, SIRT3 augments mitochondrial complex I activity, enhancing energy production. Consequently, *Sirt3*-deficient mice produce less ATP in the heart, liver, and kidney, illustrating the pivotal role of Sirt3 in energy homeostasis. In short, nutrient scarcity activates TFEB and SIRT3, increasing autophagy and mitochondrial efficiency to meet energy demands. Conversely, excess lipids in our murine *vldlr^−/−^* RAP model suppress TFEB activity, *Ppargc1a*, and *Sirt3* expression, contributing to the energy failure of photoreceptors (Figure 1).

Metabolic signals couple the energy demands of photoreceptors with adequate vascular supply. Deregulated autophagy and FAO would generate less acetyl-CoA to feed the Krebs cycle. Moreover, SIRT3-dependent inhibition of IDH2 would decrease α-KG, an essential co-factor of prolyl-hydroxylase dehydrogenase (PHD) required for the degradation of HIF1A/HIF1α (hypoxia inducible factor 1, alpha subunit). Indeed, we observe an accumulation of Krebs cycle metabolites upstream of IDH2 and a reduction of downstream metabolites, suggesting reduced IDH2 activity. Low α-KG levels are detected in human nvAMD vitreous and the murine *vldlr^−/−^* RAP model. Because HIF1A is a potent transcription factor for many pro-angiogenic genes, including VEGF (vascular endothelial growth factor), HIF1A stabilization increases angiogenesis in our model. Hence, Krebs cycle metabolites can signal to increase vascular supply to match the nutrient demands of fuel-starved photoreceptors (Figure 1). This mechanism helps explain how RAP-like neovascular lesions invade the energy- and autophagy-deficient photoreceptor layer in the murine *vldlr^−/−^* RAP model. Importantly, strategies to improve TFEB activity and autophagy, such as the *ffar1* deletion or pharmacological treatment with autophagy agonists, improve photoreceptor bioenergetics and reduce RAP-like neovascular lesions. Dysregulated autophagy by triglyceride-derived FAs might, therefore, contribute to the energy failure of photoreceptors and drive neovascular retinal diseases. We postulate that in the presence of excess dietary lipids after a meal, fatty acid sensors can send a satiety signal to protect tissue against lipotoxicity, favoring their storage, instead, in adipose tissues. However, sustained exposure to excess post-prandial lipids would reduce metabolic efficiency. In aging photoreceptors with high metabolic needs, this sustained exposure would predispose to an energy failure and trigger compensatory albeit pathological angiogenesis, leading to blinding neovascular AMD. Autophagy agonists and FFAR1 inhibition might be attractive therapeutic targets to improve neuroretinal function in neovascular AMD.
